# Charge-Transfer-Induced Reactivity in sp Carbon Atomic Wires: Toward 0‑D sp–sp^2^ Nanostructures

**DOI:** 10.1021/acs.jpcc.6c03100

**Published:** 2026-06-30

**Authors:** Marco Agozzino, Eleonora Moroni, Yifan Zhang, Valeria Russo, Carlo Spartaco Casari

**Affiliations:** † Department of Energy, Micro and Nanostructured Materials Laboratory − NanoLab, 119605 Politecnico di Milano, Via Lambruschini 8, Milano 20156, Italy; ‡ School of Engineering, Huzhou Normal University, Huzhou 313000, China

## Abstract

Carbon atomic wires (CAWs) are finite linear chains of sp-hybridized carbon atoms. Here, the electrochemical reduction of CAWs in the form of polyynes (i.e., with alternated single-triple bonds) is reported. Upon application of a reducing potential to a solution containing polydispersed hydrogen-capped polyynes, the formation of a black precipitate was observed. Electronic absorption spectroscopy confirmed the irreversible reaction of the carbon chains while excluding the degradation or side reactions. Subsequent analyses revealed that the precipitate consisted of amorphous carbon nanoparticles with a tunable diameter. This control over particle size is attributed to the modulation of growth kinetics through restricted mass transport toward the solid–liquid interface. Raman spectroscopy showed that the resulting material exhibits an amorphous sp–sp^2^ character with a retained sp fraction exceeding 60%. Smaller nanoparticles displayed reduced disorder within the sp^2^ domains, and a broader distribution of sp-chain lengths was preserved in the amorphous matrix. Additional experiments on size-selected polyynes suggest that this synthesis method allows us to better preserve the starting chain length in the final structure. Unlike previously reported amorphous sp–sp^2^ carbon networks, the nanoparticles produced in this study show remarkable stability under ambient conditions, retaining their sp character for times in excess of 6 months. These findings pave the way for future applications, particularly as further diameter tuning may enable access to the quantum dot regime.

## Graphical abstract

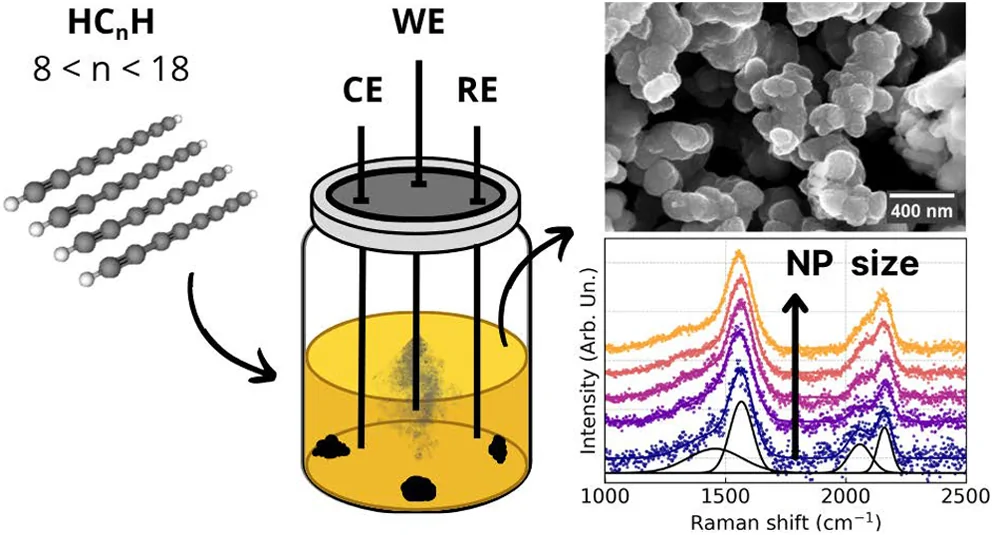

## 1 Introduction

Carbon in the sp hybridization state can be a powerful asset in the design of nanomaterials. Finite sp-carbon chains can be regarded as carbon atomic wires (CAWs) and are characterized by extensive π-conjugation. CAWs can be classified as cumulenes or polyynes, depending on whether they contain cumulated double bonds or alternating single and triple bonds.^1^ This peculiar electronic structure is key to understanding the unique optical and electronic properties of CAWs. Their strong absorption in the UV–visible range,^2,3^ exceptional hyperpolarizability,^4,5^ and large Raman scattering cross-section^6–9^ fully justify the growing interest in CAWs as prospective materials for biomedical applications,^10^ nonlinear optics,^9^ and temperature sensing.^11^ Furthermore, CAWs have been shown to effectively coordinate ions, opening the way to energy storage applications such as lithium-ion batteries and supercapacitors.^12–14^

To date, research on sp-carbon nanostructures has focused primarily on one- and two-dimensional materials. Significant efforts have been devoted to the study of CAWs as isolated chains stabilized by suitable end groups,^15^ through polymer encapsulation,^16,17^ or by insertion into nanotubes.^18^ This approach has yielded notable results, such as the fabrication of the first CAW-based field-effect transistor^19^ and electrochemical supercapacitor.^14^ It should be noted that only CAWs terminated with bulky end groups can be stabilized in the solid state, while other CAWs can only be exploited in solution.^1,15^

A notable example of two-dimensional sp–sp^2^ hybrid structures that employ sp linkers between sp^2^ domains to recreate graphene-like order is graphynes and related materials. Despite the remarkable electronic properties of these materials,^20,21^ research progress toward truly 2D atomically precise graphdiyne networks is hindered by the complex synthesis methods required and their intrinsic instability, which prevents exposure to ambient conditions.^22^

Only a few reports describe three-dimensional crystalline phases obtained through van der Waals stacking of sp-carbon chains, and the size of the ordered domains remains on the order of tens of atoms.^23,24^

More extensive progress has been achieved in the investigation of amorphous sp–sp^2^ carbon obtained through physical methods such as Supersonic Cluster Beam Deposition from a Pulsed Microplasma Cluster Source (SCBD-PMCS).^25^ However, in these studies, the sp fraction remains limited to less than 40%, and the material can only be produced in the form of nanostructured films.^6,26^ Furthermore, its stability upon exposure to air and elevated temperatures is severely limited, posing a significant obstacle to the study of its intrinsic properties and restricting potential applications.^25–27^ Still, amorphous sp–sp^2^ carbon films deposited by SCBD-PMCS under high-vacuum conditions have been stabilized and tested as electrode materials in ionic liquids, demonstrating the influence of sp-carbon chains on electrochemical double-layer capacitance.^28^ The positive influence of the sp fraction on the electrical transport in SCBD-PMCS films has also been reported, interpreting the role of the sp domains as deep defect states pinning the Fermi level out of midgap, and thus showing a self-doping effect.^26^

Quite recently, CAWs have been proposed as building blocks for novel carbon nanomaterials. In a study by Compagnini and co-workers, carbon nanowalls composed of few-layer graphene were fabricated, starting from a solution of polyynic CAWs. A 532 nm nanosecond-pulsed laser was employed to ablate a graphite target in an aqueous solution, thereby producing CAWs. During ablation, a pair of electrodes was submerged in the solution and a potential difference of 30 V was applied. The electrophoresis and subsequent assembly of the CAWs resulted in the formation of a carbon nanowall coating on the cathode.^29^ This technique has since been refined, extending deposition to polyynes synthesized via arc discharge, yielding nanowalls suitable for resistive switching memory devices.^30^ More recently, the polymerization of acetylene and the subsequent decomposition of the resulting CAWs were exploited to yield sp^2^ carbon nanoparticles with fluorescence properties.^31^

The idea of developing a bottom-up approach that relies on an sp-hybridized precursor is fascinating. However, in these works, the sp character of the reagents is lost, as the final nanomaterial consists purely of sp^2^ carbon with a high degree of graphitic order. On the other hand, it has been reported that by controlled reduction of halogenated alkenes^32–34^ and alkines,^35^ amorphous sp–sp^2^ carbon structures can be synthesized. The possibility of combining these two approaches is the starting point of this work. The main goal is then to study the reactivity of hydrogen-capped polyynes (HC*_n_* H, with 8 ≤ *n* ≤ 18), assessing whether more moderate reaction conditions can preserve the sp character in the structures that are known to form from previous studies.

Indeed, we have achieved amorphous sp–sp^2^ carbon nanoparticles with a tunable diameter. The effect of process parameters on the morphology and microstructure was investigated by Raman spectroscopy, a well-established tool for the characterization of amorphous carbon materials.^36^ The controlled reduction of size-selected polyynes was further exploited to gain insights into the reaction mechanism.

## 2 Experimental Methods

### 2.1 Polyyne Preparation

Hydrogen-capped polyynes were synthesized following a modified version of the procedure reported in ref 37. In a round-bottom flask charged with a magnetic stirring bar, 12 mL of ethanol (96%, Carlo Erba Reagents), 4 mL of DI water (σ ≤ 0.055 μS), and 20 mL of cyclohexane (≥99.5%, Sigma-Aldrich) were added. Subsequently, 0.45 g of ammonium chloride (≥99.5%, Sigma-Aldrich), 0.30 g of CuCl (≥99%, Sigma-Aldrich), 0.48 g of CuCl_2_·2H_2_O (≥97%, Sigma-Aldrich), and 0.4 g of CaC_2_ (≥75%, Sigma-Aldrich) were added in this order. The reaction mixture was stirred vigorously at room temperature for 30 min. 2 mL of concentrated HCl (37%, Supelco) was measured and brought with DI water to a total volume of 4 mL. After adding the acid solution to the reaction mixture, the organic layer was collected. A second extraction was performed with an additional 10 mL of cyclohexane. The crude product was filtered through silica (0.063–0.200 mm, Labkem) to purify it from byproducts. The refined product was transferred to acetonitrile (MeCN, ≥99.9%, Sigma-Aldrich) for electrochemical testing. Size-selected polyynes were separated by reversed-phase High-performance liquid chromatography (RP-HPLC, Shimadzu Prominence UFLC) equipped with a photodiode array (DAD) UV–vis spectrophotometer, a fraction collector (FRC-10A), and a C18 column (Phenomenex Luna 5 μm C18(2) 100 Å, LC Column 150 × 10 mm^2^). The gradient method employed is described in ref 38.

### 2.2 Electrochemical Synthesis

The MeCN solution was dried using calcium chloride (≥93.0%, Sigma-Aldrich), and tetrabutylammonium tetrafluoroborate (TBA-TFB, ≥99.0%, Sigma-Aldrich) was added as a supporting electrolyte. The concentration of HC_8_H, the most abundant polyyne in the mix, was determined from UV–visible spectra acquired with a Shimadzu UV-1800 spectrophotometer. A molar extinction coefficient ε = 199,550 L mol^–1^ cm^–1^ was used for the absorption band at 225 nm.^2^

Measurements were conducted in a three-electrode cell employing Pt wires (250 μm, 99.99%, Goodfellow Cambridge Ltd.) as working, counter, and reference electrodes. The sample volume in the cell was fixed at 3 mL. To study deposition on the electrode surface, gold-coated silicon electrodes were fabricated using a Edwards E306 thermal evaporator. 45 nm Au (99.9%, Mateck) films were deposited on Si wafers (test grade, Siegert Wafer), adding a 5 nm titanium (97.5% purity, Sigma-Aldrich) adhesion-promoting layer. Electrochemical measurements were conducted on a Palmsens 4 potentiostat. For each sample, after a preliminary cyclic voltammetry measurement, the potential at the working electrode was kept constant at −1.8 V vs Pt pseudoreference electrode for 400 s.

### 2.3 Structural and Morphological Characterization

After electrochemical measurement, each sample was allowed to rest and a black precipitate was collected at the bottom of each vial. The precipitate was collected and rinsed with fresh acetonitrile to remove leftover polyynes and electrolyte. The clean precipitate was deposited on a silicon substrate for further characterization. Scanning electron microscopy (SEM) images were collected using a Zeiss Supra 40 field emission SEM, keeping the accelerating voltage at 5 kV to avoid damaging the sample. Raman spectra were collected through a Renishaw InVia microRaman spectrophotometer equipped with an Ar^+^ laser emitting at 514 nm and a 1800 lines/cm grating. Power-on sample was set at 0.05 mW, increasing up to 0.25 mW for low-signal samples.

## 3 Results and Discussion

The first evidence of the peculiar electrochemical behavior of polyynes in solution comes from cyclic voltammetry (CV). From Figure 1a, it is evident that the addition of polydispersed H-capped polyynes greatly increases the overpotential required for the solvent degradation. The redox peaks observed around −1.5 V vs Pt can be attributed to the oxygen dissolved in MeCN.^39^ This reactivity is quenched by the addition of CAWs. Furthermore, when reaching the lower edge of the imposed potential window, a black precipitate can be seen to form around the working electrode. By applying a negative potential (−1.8 V vs Pt) for 400 s, the amount of precipitate further increases. The electronic absorption spectra reported in Figure 1b further confirm the consumption of polyynes upon the electrochemical reduction. Quite interestingly, the background usually associated with degradation byproducts is not observed, and the relative decrease in concentration is found to be uniform for all chain lengths (as can be seen from the inset of Figure 1b). The different values obtained for the case of HC_18_H can be ascribed to the uncertainty deriving from its low concentration.

**Figure 1:**
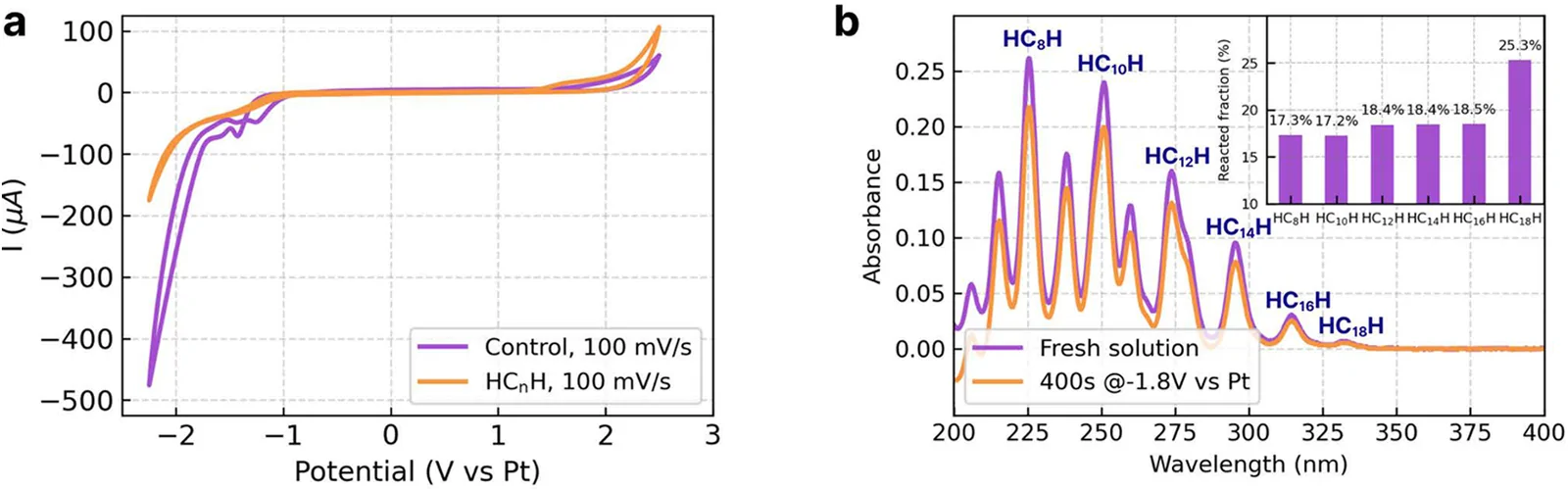
Electrochemical synthesis (a) Cyclic voltammogram of polyynes in anhydrous MeCN with 0.1 M TBA-TFB as the supporting electrolyte. The CV of dry MeCN is shown as a reference. (b) Electronic absorption spectra of polyynes in MeCN before and after a potentiostatic run. In the inset, the polyyne consumption is reported. It is expressed as the fraction of the initial concentration, and it has been determined from the intensity variation of the main vibronic peak for each chain length.

The results obtained by CV are consistent with the formation of a new compound at the electrode surface, which, by hindering mass and electron transport, results in sluggish degradation kinetics for the acetonitrile TBA-TFB solution. By collecting the precipitate and performing SEM imaging, it was found that the reduction of polyynes in solution yields a dispersion of nanoparticles. Remarkably, by changing the concentration of polyynes in the solution, it was observed that the size distribution could be tuned. A similar effect was observed upon a reduction in the supporting electrolyte concentration, as is evident from the SEM images in Figure 2a–e. The average diameter and standard deviation obtained from the different experimental runs are reported in Figure 2f.

**Figure 2:**
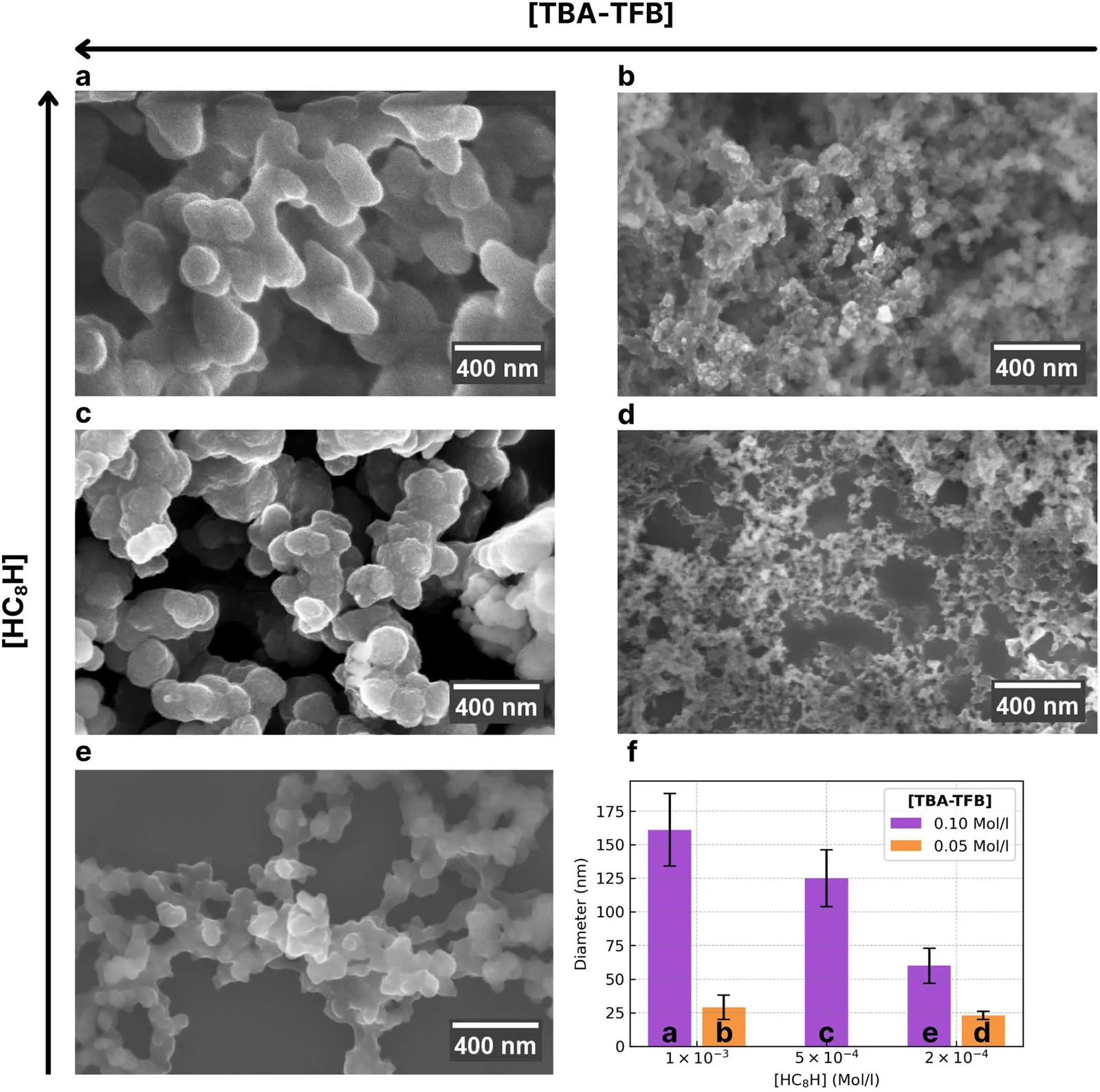
Morphological characterization scanning electron microscopy (SEM) images of nanoparticle aggregates obtained after a 400 s potentiostatic run at −1.8 V vs Pt pseudoreference from mixed CAWs in ACN solution with a supporting electrolyte concentration of 0.1 M and an HC_8_H concentration of (a) 1 × 10^–3^ M, (c) 5 × 10^–4^ M, and (e) 2 × 10^–4^ M. A second set of experiments was performed with a supporting electrolyte concentration of 0.05 M and an HC_8_H concentration of (b) 1 × 10^–3^ M and (d) 2 × 10^–4^ M. Higher resolution SEM images of the last two samples can be found in Figure S1. The corresponding size distributions are summarized in (f).

A straightforward interpretation of this phenomenon can be found by considering that the size of the nanoparticles generated at the electrode surface is a result of the balance between growth and nucleation. In this instance, the diameter of the nanoparticles is governed by the density of polyynes available to react at the solid–liquid interface. By limiting the precursor concentration in the bulk solution, a shallower gradient is established in the diffusion layer, thus hindering mass transport toward the electrode and slowing down growth.

The effect of the supporting electrolyte is analogous; by enhancing the solution’s conductivity, the reaction rate can be increased as the Ohmic drop is reduced, promoting nanoparticle growth.

Raman spectroscopy proved to be a valuable asset in understanding the structure of the nanoparticles. The spectra reported in Figure 3a are characterized by the spectral features of amorphous sp–sp^2^ carbon. Both the G and D bands, usually associated with sp^2^ carbon, can be discerned, even though the latter is quite weak and broad. Interestingly, a quite intense band is also present in the region between 1900 and 2200 cm^–1^. This spectral feature is peculiar to materials that incorporate sp carbon chains.^6,26–28^ As commonly observed in other disordered sp–sp^2^ structures, the vibrational modes give rise to broad bands. Moreover, two components can be discerned in the sp band (henceforth referred to as sp′ and sp″, respectively). For this reason, two Gaussians were used to fit the features associated with sp carbon. Two Gaussians were also used to fit the D and G bands. This procedure is less informative than the more established approach that describes the D band with a Lorentzian and the G band with a Breit-Wigner-Fano (BWF) function.^36^ However, the noise in the spectra renders impractical the implementation of a BWF-Lorentzian approach. Indeed, the D band contribution is captured by the asymmetry in the BWF, resulting in fitting parameters that are nonphysical. A full overview of the spectra processed in this work, as well as the results from the fitting procedure, can be found in Figure S2 and in the code shared together with the raw data.

**Figure 3:**
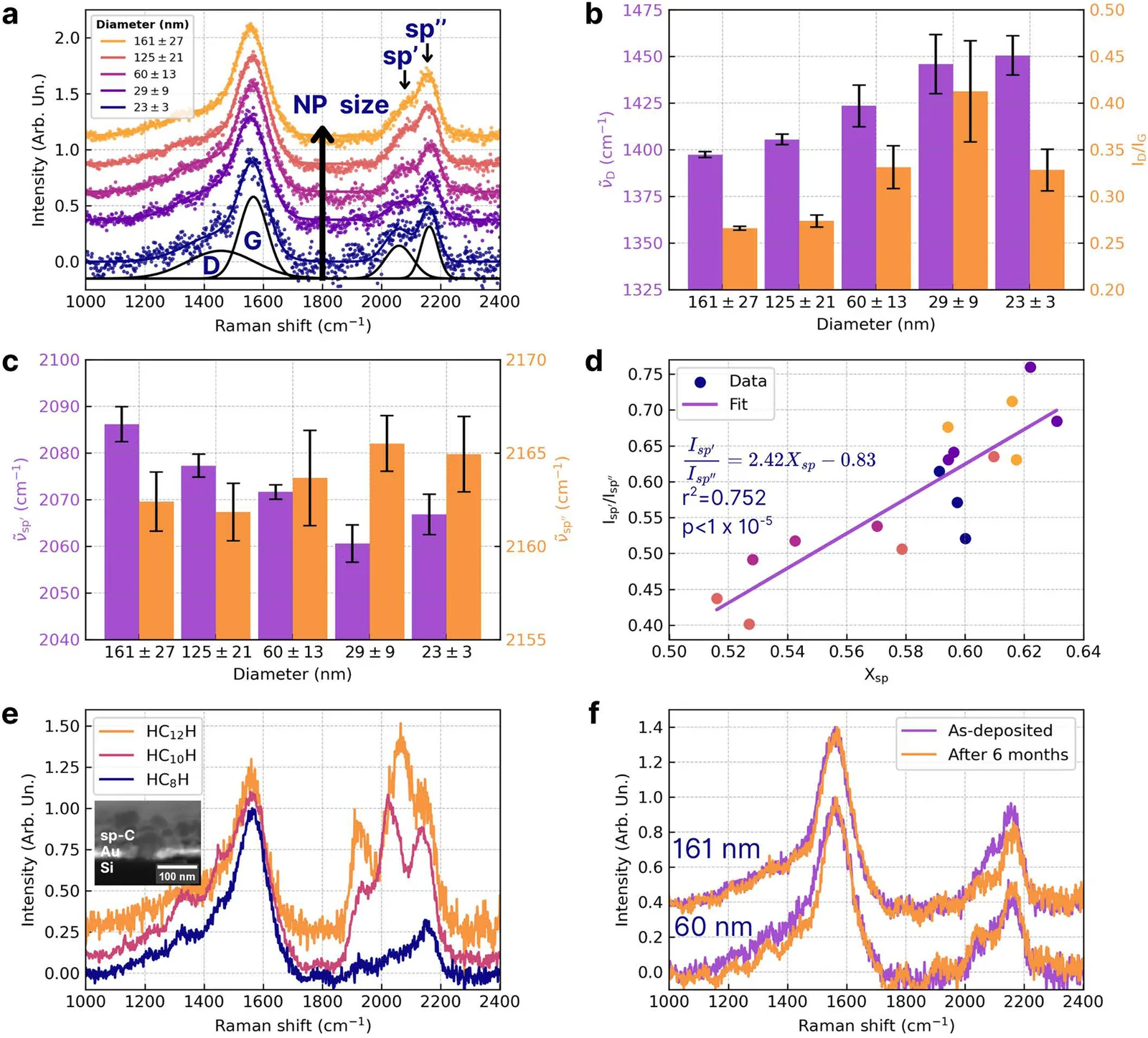
Spectroscopic characterization. (a) Baseline-corrected Raman spectra of nanoparticle aggregates obtained after a 400 s potentiostatic run at −1.8 V vs Pt pseudoreference from mixed CAWs in ACN solution. (b) D-line Raman shift and intensity ratio between the D and G bands. (c) Raman shift of the sp′ and sp″ bands. (d) Correlation between the sp fraction and intensity ratio between the two sp components. These parameters were retrieved from the spectra in (a). (e) Raman spectra of a Au-coated Si electrode (SEM image shown in the inset) after a potentiostatic run in an ACN solution containing size-selected CAWs. (f) Spectra of the nanoparticles with *d* = 161 ± 27 nm and *d* = 60 ± 13 nm after drying and after 6 months stored in ambient condition, protected from light exposure. For all Raman spectra, the excitation wavelength is 514 nm.

The evolution of the sp^2^ component in this material with decreasing nanoparticle dimensions can be traced by considering Figure 3b and, more precisely, the intensity ratio between the D and G bands (*I*_D_/*I*_G_). It is well known that this metric can be reliably related to the degree of disorder in sp^2^ domains, as it increases with the degree of disordering. This trend is held up to a threshold after which it is reversed, as a further disordering of the structure results in the disgregation of the six-membered rings that are necessary for the D band signal.^36^

It is reasonable to assume that the material studied in this work lies in this last stage of amorphization, and thus an increasing *I*_D_/*I*_G_ ratio for smaller nanoparticles can be interpreted as a possible sign of a more ordered structure. While the center of the G band does not vary significantly, a blue shift of the D band frequency ν̃_D_ is observed. Even if there is no clear consensus on the physical interpretation of the shift in ν̃_D_, it has been proposed that higher frequencies are linked to ordering in highly disordered carbon structures, where the D band also appears at unusually large energies, as is the case in this work.^40^ This, together with the band’s large width, suggests a point of caution as it has been pointed out that in highly inhomogeneous structures the interpretation can be more nuanced than just considering disordering of the graphene-like network. Indeed, structural heterogeneity, interfacial coupling, and functionalization can modulate the Raman spectrum through resonance enhancement, charge transfer, strain, and sp^3^ bonding.^41^

For this reason, it is worth noting that the D line shift is coherent with the indication extracted from the ratio *I*_D_/*I*_G_ and thus can be related to the mechanistic interpretation we have hypothesized for the influence of precursor and electrolyte concentration on the resulting nanoparticle size. It can be inferred that the slower growth kinetics of smaller nanoparticles is reflected in a more ordered structure at the atomic scale.

More challenging is the discussion regarding the information that can be extracted from the signal associated with the sp carbon.

CAWs can host a collective oscillation along the chain, often referred to as the Effective Conjugation Coordinate (ECC) mode. The ECC is Raman active, and its frequency falls in the region between 1800 and 2100 cm^–1^. Polyynic CAWs exhibit a higher-frequency ECC (i.e., typically higher than 2100–2200 cm^–1^), while cumulenic CAWs tend to present Raman signals at lower wavenumbers. In both cases, longer chains typically imply a more relaxed ECC mode.^1^ Theoretical studies on the formation of cluster-assembled films suggest that sp–sp^2^ carbon structures are formed by disordered sp^2^ domains encapsulating polyynic sp carbon chains with different lengths.^42,43^ It must be noted that these results consider growth in the plasma phase and thus may be of limited validity to the present work, where it would be more realistic to hypothesize that the sp chains act as linkers between sp^2^ aggregates. Calculations^44^ also suggest that the sp band observed in amorphous film can be deconvolved as the sum of several signals originating from the coexistence of chains of different lengths and with different curvature and torsional angles. Obviously, this model is somewhat simplified, and other factors such as strain could also play a role. Due to the small amount of material achieved in this work, it was only possible to reliably discern two components (sp′ and sp″), as mentioned earlier. Nonetheless, if we assume the main contribution to the signal frequency to be from chain length, the shift in ν̃_sp′_ and ν̃_sp″_ evident from Figure 3c can be interpreted as a broadening of the length distribution of the sp linkers in the amorphous structure. A slower reaction kinetics would then not only result in smaller nanoparticles but would also allow for better preservation of the chain length distribution present in the precursor solution. Within this framework, *I*_sp′_/*I*_sp″_ can be taken as a qualitative indicator of the ratio between the number of longer and shorter chains. If we assume that a larger fraction of longer chains in the amorphous matrix (i.e., larger *I*_sp′_) will be responsible for a higher overall number of sp-carbon atoms, *I*_sp′_/*I*_sp″_ should correlate to the fraction of sp-hybridized carbon atoms *X*_sp_. Now, by assuming as a zero-order approximation a linear relationship between *X*_sp_ and *I*_sp′_/*I*_sp″_, we can fit the data in Figure 3d. Indeed, a correlation seems to hold between these two parameters, suggesting *I*_sp′_/*I*_sp″_ as a qualitative indicator of the sp carbon content in amorphous sp–sp^2^ carbon materials.

The fraction of sp-hybridized atoms has been evaluated following the method proposed by D’Urso and co-workers for cluster-assembled films.^45^ By postulating that the structure of the material studied in this work is similar to what was achieved by D’Urso and thus that the sp^3^ content is negligible, eq 1 holds, where the ratio between the scattering cross sections is σ_sp_/σ_sp^2^_ = 0.48 for the 514 nm excitation wavelength.

1 $$\frac{X_{\mathrm{sp}}}{X_{sp^{2}}}=\frac{I_{\mathrm{sp}}}{I_{sp^{2}}}\cdot \frac{\sigma_{sp^{2}}}{\sigma_{\mathrm{sp}}},\mathrm{where}\;X_{\mathrm{sp}}+X_{sp^{2}}=1$$

By evaluating the area under the sp and sp^2^ bands (*I*_sp_ and *I*_sp^2^_), the fractions of sp and sp^2^ hybridized atoms (*X*_sp_ and *X*_sp^2^_) can be retrieved. The average values for the different experimental runs are reported in Table 1. It must be noted that a more conservative estimation of the ratio σ_sp_/σ_sp^2^_ has been proposed by Ravagnan and co-workers, even though Raman and near-edge X-ray absorption fine structure spectroscopy measurements were conducted on separate samples.^6^ It would be interesting to perform synchrotron-based measurements on sp–sp^2^ carbon synthesized through electrochemical methods to better assess the proposed similarity with the structure of films deposited via physical techniques.

**Table 1:** Average sp Carbon Content Extracted from the Raman Spectra

diameter (nm)	161 ± 27	125 ± 21	60 ± 13	29 ± 9	23 ± 3
*X*_sp_ (%)	59.6 ± 0.4	61.1 ± 1.6	54.7 ± 1.7	55.8 ± 3.8	60.9 ± 1.1

To further investigate the mechanism behind the formation of nanoparticles, the same electrochemical process was performed on size-selected polyynes, namely, HC_8_H, HC_10_H, and HC_12_H. Figure 3e shows the Raman spectra acquired on the electrode surface (a SEM micrograph is shown in the inset). Clearly, longer chains yield a material with a stronger sp Raman fingerprint and result in a shift of the sp band toward lower frequency. Following the analysis proposed in ref 44, it is not possible to directly link each of the components in the spectra to a single chain length based on the frequency of the ECC mode of H-capped polyynes. However, the range is coherent with the presence of sp linkers between 12 and 2 atoms. This suggests a possible interpretation: charge transfer could be responsible for the activation of the hydrogen end groups, thus favoring chain termination reactivity. This, in turn, would result in the preservation of the starting chain length in the final amorphous network. Thus, starting from a solution containing longer polyynes would result in nanoparticles with a larger fraction of sp-carbon atoms.

A final remark can be made about the stability of the sp component in the nanoparticles. As evidenced from the Raman spectra in Figure 3f, there is a relatively small decrease in the signal associated with the sp component even after 6 months of exposure to ambient conditions. This is quite remarkable as previous examples of amorphous sp–sp^2^ carbon are only stable under high vacuum and for a limited time.^25^ It is possible that the electrochemical synthesis route is able to passivate reactive sites that would otherwise induce the degradation of the sp linkers toward more stable sp^2^ domains. This mechanism would also explain the apparently higher stability shown by smaller nanoparticles as they are generated by slower kinetics, likely linked to better passivation. While this enhancement in stability opens a new possibility to implement sp carbon nanostructures in future devices, a more detailed characterization is needed. The exposure to aggressive chemical agents as well as high temperature in an oxidizing atmosphere are all conditions that must be tested in future studies.

## 4 Conclusions and Outlook

In this work, the charge-transfer-induced reactivity of polyynic carbon atomic wires was explored. The electrochemical reduction of polydispersed H-capped polyynes in solution was demonstrated to yield amorphous carbon nanoparticles. The diameter was found to be tunable by suitably selecting the precursor and supporting electrolyte concentration. Raman spectroscopy highlighted the significant presence of sp-hybridized carbon atoms in the final structure.

This phenomenon was attributed to the reductive activation of the hydrogen end groups, which is hypothesized to preserve the chain length during the cross-linking process. This hypothesis was further supported by analyses performed on the size-selected polyynes. Raman spectroscopy also allowed the investigation of structural disorder in the amorphous nanoparticles, revealing that smaller nanoparticles are characterized by an ordered sp^2^ network containing a broader range of sp carbon chains.

Unlike previous examples of amorphous sp–sp^2^ carbon obtained through physical methods, the electrochemical process explored in this study yielded a material that is stable in air and can be tested ex situ, opening opportunities for future studies toward a deeper understanding of the physics underlying this class of materials with strong application potential.

However, many aspects of this novel structure remain unclear. For instance, the mechanism behind the increased stability achieved through electrochemical methods needs to be further clarified. Additional studies are also required to investigate the effect of other process parameters, most importantly the applied potential: more negative potentials could increase the nucleation rate, further decreasing nanoparticle size. With suitable optimization, the diameter could potentially be reduced to the point of inducing quantum dot behavior. Furthermore, in this work, noble metal electrodes were used; however, the electrode material itself may play an unexplored role in polyynes’ reactivity.

## Supplementary Material



## Notes

High-resolution SEM images and fitted Raman spectra of the samples (PDF)
