# Supplementary material for: Charge-Transfer-Induced Reactivity in sp Carbon Atomic Wires: Toward 0‑D sp–sp2 Nanostructures
Source: J Phys Chem C Nanomater Interfaces. 2026 Jun 30;130(42):14588–95. doi: 10.1021/acs.jpcc.6c03100 (PMC13701073; doi:10.1021/acs.jpcc.6c03100)
Supplement: Supplementary file 1 [file jp6c03100_si_001.pdf]

Supporting Information for the manuscript

Charge-Transfer Induced Reactivity in *sp* Carbon Atomic Wires: Towards 0–D *sp*–*sp*² Nanostructures

Marco Agozzino[a], Eleonora Moroni[a], Yifan Zhang[a,b], Valeria Russo[a], and Carlo Spartaco Casari*[a]

[a]Department of Energy, Micro and Nanostructured Materials Laboratory – NanoLab, Politecnico di Milano, Via Lambruschini 8, Milano, 20156, Italy
[b]School of Engineering, Huzhou Normal University, Huzhou, 313000, China

*Corresponding author

a b

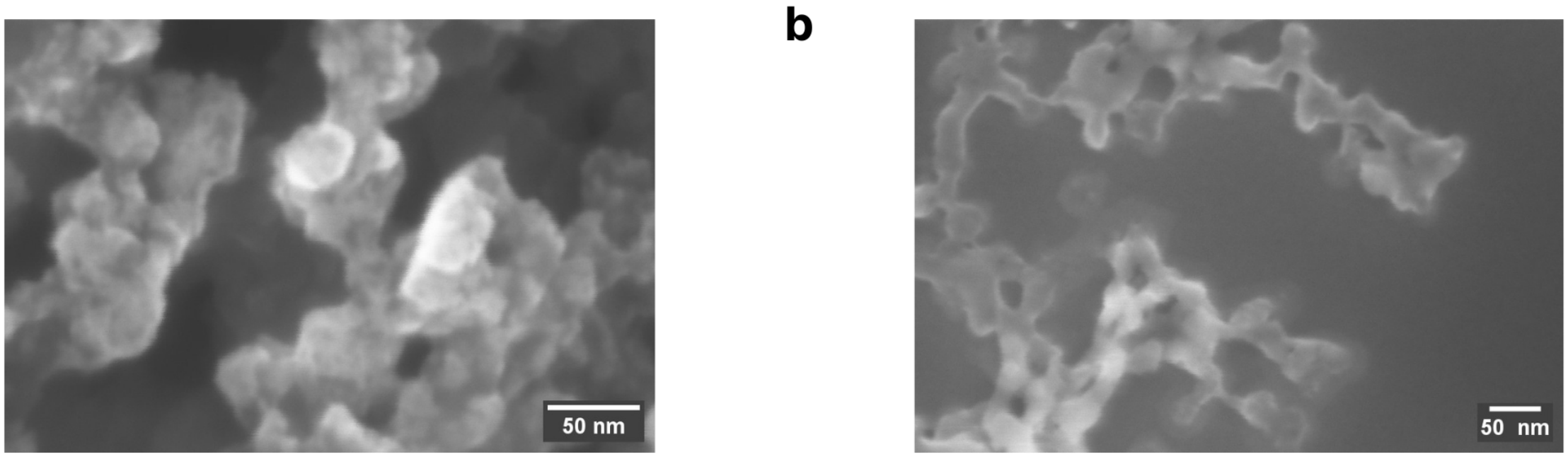

Figure S1: High magnification SEM images of the nanoparticles obtained from mixed CAWs in ACN solution with a supporting electrolyte concentration of 0.05 M and an HC_8H concentration of **a)** 1×10^{-3} M and **b)** 2×10^{-4} M.

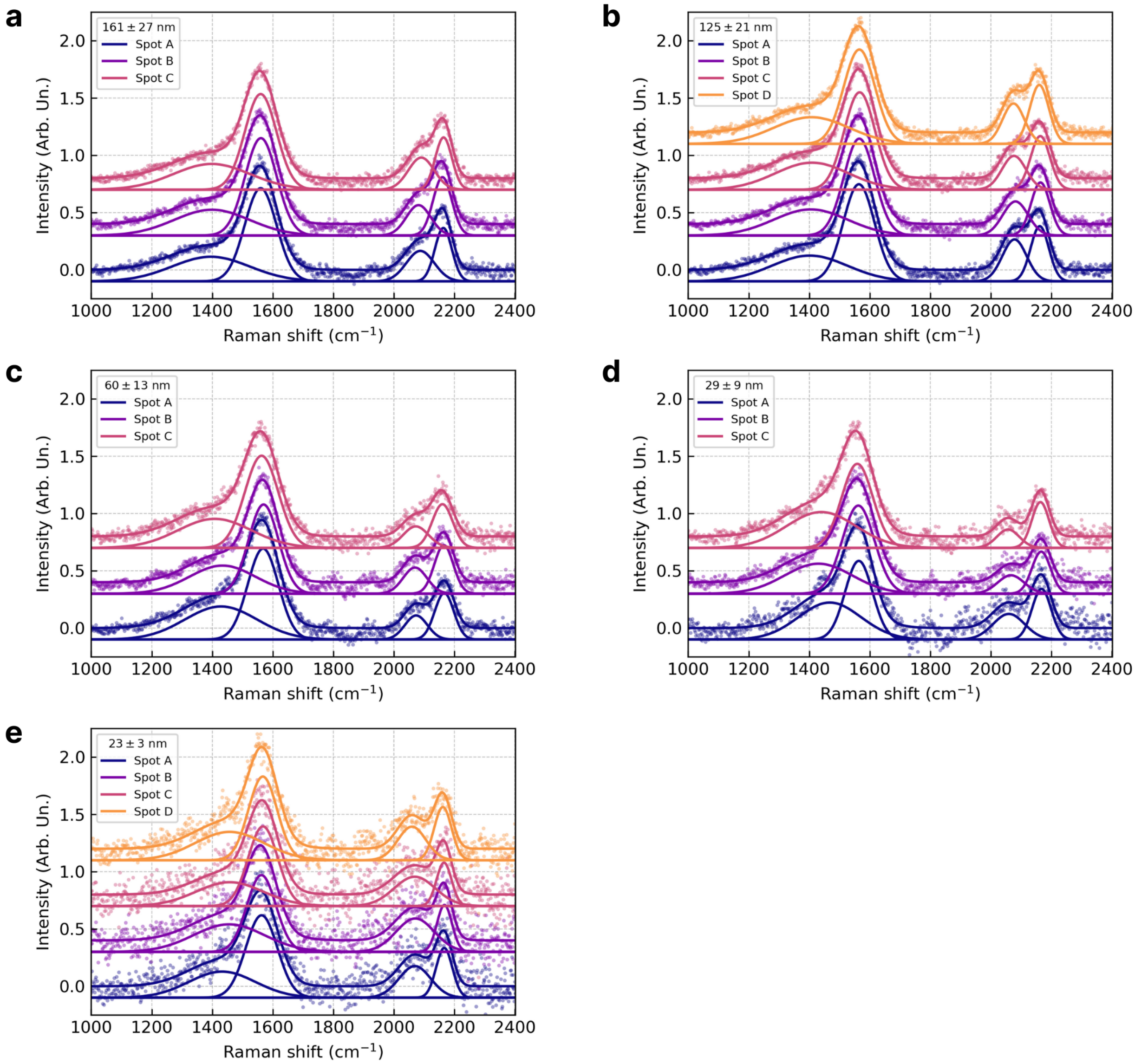

Figure S2: Baseline-corrected Raman spectra obtained from the different experimental runs plotted together with the fitted curve and single gaussian contributions. For a complete overview of the fitting parameters, the reader can refer to the code shared together with the raw data in the repository `https://doi.org/10.5281/zenodo.19628676`.
